# Enhanced Wear Resistance of Tungsten-Reinforced Brass Surface Composite Produced Through Friction Stir Processing at Varying Tool Rotational Speed

**DOI:** 10.3390/ma19091745

**Published:** 2026-04-24

**Authors:** Haitham M. Alswat, Karpagarajan Sivaraman, Balamurugan Chinnasamy, Vigneshwaran Soundararaja Perumal, El-Sayed I. Abdel Aziz

**Affiliations:** 1 Department of Mechanical Engineering, College of Engineering, Shaqra University, Riyadh 11911, Saudi Arabia; halswat@su.edu.sa; 2 Department of Mechanical Engineering, Dhanalakshmi Srinivasan Engineering College, Perambalur 621212, Tamil Nadu, India; 3 Department of Mechanical Engineering, College of Engineering Guindy, Anna University, Chennai 600025, Tamil Nadu, India; balamurugan.c@annauniv.edu; 4 Department of Mechanical Engineering, Faculty of Engineering and Technology, SRM Institute of Science and Technology, Ramapuram Campus, Chennai 600089, Tamil Nadu, India; svigneshwaranmech2010@gmail.com; 5 Material Engineering Department, Faculty of Engineering, Zagazig University, Zagazig 44519, Egypt; engsayed@su.edu.sa

**Keywords:** friction stir processing, brass, surface composite, sliding wear behavior, tungsten particle, hardness, microstructure, wear resistance, tool rotational speed, adhesion wear

## Abstract

This study examines the effect of tool rotational speed on the microstructure and dry sliding wear behavior of brass–tungsten (brass/W) surface composites fabricated through friction stir processing. Microstructural analysis confirmed a uniform distribution of tungsten particles within the stir zone, with no observable clustering. Improved properties were achieved at a lower traverse speed of 40 mm/min combined with a higher rotational speed of 1168 rpm, which promoted finer grain formation (~4 µm) and better particle dispersion. An increase in rotational speed led to a corresponding rise in hardness, from 142 HV at 832 rpm to 165 HV at 1168 rpm. In terms of wear behavior, the sample processed at lower rotational speed exhibited abrasive and micro-cutting wear, whereas the sample processed at higher rotational speed predominantly showed adhesive wear.

## 1. Introduction

Brass is a copper-based alloy that is commonly used due to its excellent strength, machinability, and corrosion resistance. The advantageous properties of brass have led to its widespread use in various industrial components, including thermal heat exchangers, electrical gears, transportation equipment (such as bearings and bushes), swash plates, and fuel systems [1]. However, brass is susceptible to gradual sliding wear during its service life [2]. In order to overcome wear, surface composites can be developed on brass. This method economically permits the preservation of toughness. Brass surface composites enhance service life and provide a suitable performance–economic balance [3]. Multiple methods have been used to produce bulk as well as surface composites with different kinds of reinforcements. A few commonly adopted methods for the production of composites are the powder metallurgical route [4], electro-deposition [5], and stir casting [6]. In spite of these available methods, using any of these methods for brass composite production causes a loss of zinc during processing due to melting and vaporization. Moreover, porosity and clustering of reinforcement particles deteriorate the quality of brass composites [3,7,8].

Friction stir processing (FSP) is a solid-state method that is excellent for producing surface composites, which can overcome the challenges of other methods [3]. FSP is advantageous for producing various combinations of matrix and reinforcement without melting. The frictional heat produced by the FSP tool plasticizes and forges the matrix as well as reinforcement at the back end as the tool traverses. The major advantage of this process is that entrapment of atmospheric gases and formation of porosity are largely eliminated. The modest FSP operating temperature blocks the formation of undesirable chemical reactions in the matrix–reinforcement interface and this paves a path to hold FSP as a prolific method for preparing surface composites [9]. The deformation produced by FSP ensures uniform dispersion of reinforcement in the matrix, which is a critical factor in achieving the desired improvement in material properties [10,11]. The deformation and heat due to friction modify the microstructure and restrict the development of recrystallized grains, resulting in a fine-grained microstructure. In addition, the parameters, namely the volume fraction of reinforcement, tool rotational speed, and traverse speed, show a notable influence on the structure–property relationships of fabricated surface composites [12]. Surface composites produced via FSP record notable increases in strength and resistance to wear and corrosion [13]. One study on a brass/graphite composite showcased better reinforcement distribution when recursive FSP was executed, which finally depicted improved material properties [14]. Heidarzadeh et al. [15] fabricated a brass/Al_2_O_3_ surface composite using FSP and documented the interaction of zinc and atmospheric gases. Moreover, a further understanding of the preparation of brass surface composites provides valuable insight into the preparation of surface composites using FSP. Besides, the effect of tool rotational speed on the structure–property relationships of brass surface composites is lagging. Past studies deployed various reinforcements like organic, inorganic, fibrous, and metallic particles for the fabrication of composites using FSP, and among the reinforcements used, the metallic reinforcements exemplified promising results [16].

Although FSP has been widely employed for the fabrication of surface composites, studies focusing on brass-based systems reinforced with tungsten particles are relatively limited. In particular, the influence of tool rotational speed on the dispersion behavior of tungsten and its subsequent effect on microstructural evolution and tribological performance has not been comprehensively addressed. Moreover, the combined use of advanced characterization techniques such as electron backscatter diffraction (EBSD) and transmission electron microscopy (TEM) to understand grain refinement mechanisms and reinforcement–matrix interactions remains scarce.

In this context, the present work aims to investigate the fabrication of brass/W surface composites using friction stir processing over a range of tool rotational speeds. Special emphasis is placed on understanding the distribution of tungsten particles, grain refinement mechanisms, and their influence on hardness and wear behavior. The integration of EBSD and TEM analyses provides deeper insight into the structure–property relationships governing the performance of the developed composites.

This study contributes to both the fundamental understanding and practical application of friction stir processed surface composites. From a scientific viewpoint, it clarifies the role of tool rotational speed on grain refinement, tungsten particle distribution, and the resulting changes in hardness and wear behavior. The combined use of EBSD and TEM provides insight into the mechanisms governing microstructural evolution. From an application perspective, the developed brass/W surface composite shows improved hardness and wear resistance, making it suitable for components exposed to sliding conditions. The approach offers a feasible method to enhance surface performance without modifying the bulk material, which is beneficial for extending component service life in industrial applications.

## 2. Experimental Procedure

The original dimensions of the procured brass plate measured 100 × 50 × 10 mm^3^. The brass plate was procured in India and it exhibited the following chemical composition: 40 wt.% Zn, 1.56 wt.% Pb, 0.48 wt.% Sn, 0.31 wt.% Fe, 0.25 wt.% Ni, 0.04 wt.% Al, 0.03 wt.% Sb, 0.01 wt.% Mn, and 0.01 wt.% Si, with the remaining composition consisting of copper (Cu). A keyway was machined into the brass plate, featuring dimensions of 5 mm in depth and 1.2 mm in width. The W particles (approximately 5 µm in size), making up approximately 9% by volume, were used as reinforcement. The keyway was subjected to processing using a flat cylindrical tool designed for FSP, constructed from high-carbon–high-chromium (HCHCr) steel. The FSP procedure was carried out utilizing a CNC vertical milling center. The hexagonal pin-shaped FSP tool had a 6 mm pin diameter and a 5.7 mm pin length with a 24 mm shoulder diameter. Before the execution of FSP, the tool was tempered twice. At the start of FSP, a pinless tool was deployed for the capping operation to restrict the escape of W particles. The rotational speed of the FSP tool varied at 832, 900, 1000, 1100, and 1168 rpm while the traverse speed (s) remained fixed at 40 mm/min, and the volume fraction (v) of W particles was maintained at 9%. Previous studies [2,8] on friction stir processed surface composites have reported that reinforcement levels in the range of 5–10 vol.% provide a suitable balance between uniform particle distribution and property enhancement. In addition, lower traverse speed increases the interaction time between the tool and material, resulting in sufficient frictional heat generation, improved plasticization, and effective redistribution of particles within the stir zone. On the other hand, the higher traverse speeds were found to reduce heat input and limit material flow, which can lead to inadequate mixing and possible particle agglomeration. Therefore, a constant traverse speed of 40 mm/min was chosen to maintain stable processing conditions while investigating the effect of tool rotational speed as the primary variable. The actual volume fraction of W particles was determined using the relation ${(V}_{A})$= $\left(\frac{A_{g}}{A_{s}}\right)\times 100$, where *V_A_* is the actual volume fraction of W particles (in %), *A_g_* is the keyway area (in mm^2^) and *A_s_* is the area of the surface composite (in mm^2^). In the geometric relation of *A_g_* = *W_g_* × *d_g_*, *W_g_* denotes keyway width and dg indicates keyway depth (all in mm). Previous studies on FSPed surface composites have reported that reinforcement levels in the range of 5–10 vol.% provide a suitable balance between uniform particle distribution and property enhancement. A lower traverse speed increases the interaction time between the tool and material, resulting in sufficient frictional heat generation, improved plasticization, and effective redistribution of tungsten particles within the stir zone. Based on these, the process parameters were from a previous study [2] and experimental trials. Prior to commencing FSP operations, a flat tool was used to cap the keyway, preventing the runaway of W metallic particles. The tilt angle of the FSP tool was 0° and the tool was traversed one pass to create the surface composite. To assess the effects of these procedures, specimens for metallographic examination were drawn out from the processed region of the brass plates, following established metallographic techniques. For micro and macrostructural analysis, the samples were etched in a solution containing 15 mL HCl, 2.5 g FeCl_3_, and 100 mL distilled water. Microstructural features were revealed using a color etchant composed of 24 g sodium thiosulfate, 2.4 g lead acetate, and 3 g citric acid in 100 mL distilled water. Macrographs and micrographs were captured using an Olympus SZX16 stereo microscope (Olympus, Tokyo, Japan) and an Olympus BX51M optical microscope (Olympus, Tokyo, Japan), respectively. Macrographs and micrographs were captured using optical microscopes. Grain orientation was analyzed using EBSD after electropolishing the BMSCs/W samples for 20 s in a solution of 20% perchloric acid and 80% methanol using a Struers Electropol-IV unit. Misorientation maps were obtained using FEI-Quanta 3D FEG-SEM (FEI Company, Eindhoven, The Netherlands) with a 0.3 µm step size and processed using TSL OIM 4.6 software. For TEM analysis, thin foils (~100 µm) were prepared by twin-jet electropolishing in a standard electrolyte at −30 °C and examined using a JEOL JEM 2100 TEM at 200 kV (JEOL, Tokyo, Japan). Microhardness measurements were carried out using a Mitutoyo HM-220 microhardness tester (Mitutoyo, Kanagawa, Japan) with an applied load of 500 g and a dwell time of 15 s. Indentations were taken across the processed region, covering the stir zone, thermo-mechanically affected zone, and base material over a total distance of 28 mm. For each condition, five measurements were recorded at different locations, and the average value was considered for analysis. Wear specimens (4 × 6 × 20 mm^3^) were prepared from the stir zone using wire-cut EDM. Dry sliding wear tests were conducted on a DUCOM TR20-LE pin-on-disk setup (Ducom Instrument, Bengaluru, India) under ASTM G99-04 [17] conditions at 30 N load, 1.5 m/s velocity, and 2500 m sliding distance. For each condition, a minimum of three trials was performed to ensure repeatability, and the average value was reported. Wear rate was calculated from pin height reduction using $K=\frac{v}{L\times d}$, where *v* = wear volume loss (mm^3^), obtained from the reduction in pin height, *L* = applied normal load (N) and *d* = sliding distance (m) and the average of the three tests was reported. Worn surfaces and collected debris were analyzed using SEM.

Furthermore, hardness measurements were obtained using a microhardness testing unit, covering the entire cross-sectional area. Samples obtained from the stirred zone were utilized for wear studies. Under dry conditions, sliding wear tests were conducted within a pin-on-disk unit, applying a regular force of 30 N, 1.5 m/s of sliding velocity and 2500 m of sliding distance. The wear rate was determined based on the measured reduction in pin height. Subsequently, the worn samples underwent scanning electron microscopy (SEM) analysis to investigate surface topography and wear mechanisms.

## 3. Results and Discussion

### 3.1. Effect of Tool Rotational Speed on Microstructure

**Figure 1:**
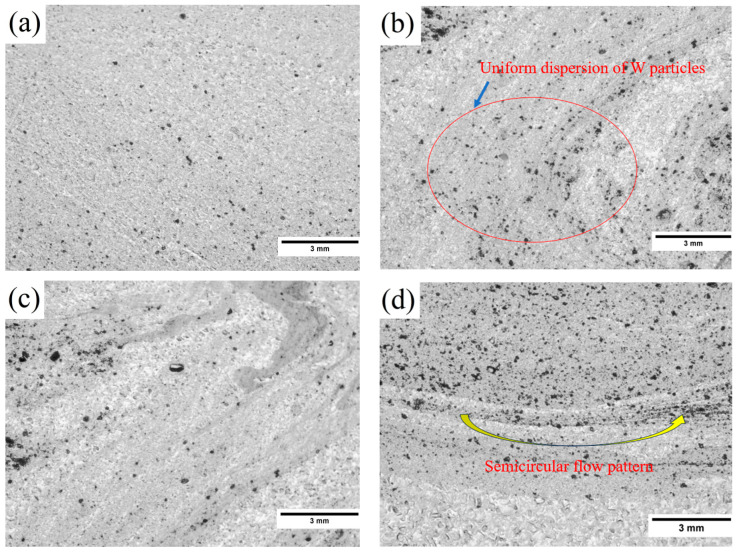
Optical images of brass/W illustrating the distinct regions within the stir zone, including: (**a**) near the top substrate; (**b**) retreating portion; (**c**) advancing portion; (**d**) bottom section.

The optical microstructures of brass/W are depicted in Figure 1a–d, and the micrographs indicate the successful formation of the composite without any noticeable defects. Additionally, there are no visible remnants of the previously machined keyway. As shown in Figure 1d, the heat generated during the FSP process softens the brass below the stir zone and changes the shape significantly. The rotation of the FSP tool helps the compacted W particles to spread out in the pliable matrix. Following this, the material flow is effectively consolidated, resulting in the formation of a brass/W composite in the stir zone. The stir zone size is proportional to the area of the fabricated surface composite. The stir zone width is noted to be larger than the diameter of the tool pin, which may be due to the relatively high thermal conductivity of brass. This characteristic leads to a broader dispersion of frictional heat. Moreover, lower traverse speed increases the frictional heat that enlarges the plasticizing region of brass. However, the selected process parameters and FSP tool morphology resulted in a semicircular flow pattern beneath the stir zone rather than the formation of any onion ring pattern. This semicircular flow pattern is an indication of the possible formation of onion rings, and the contribution of the FSP tool is more in the formation of recognizable semicircular onion rings [18].

**Figure 2:**
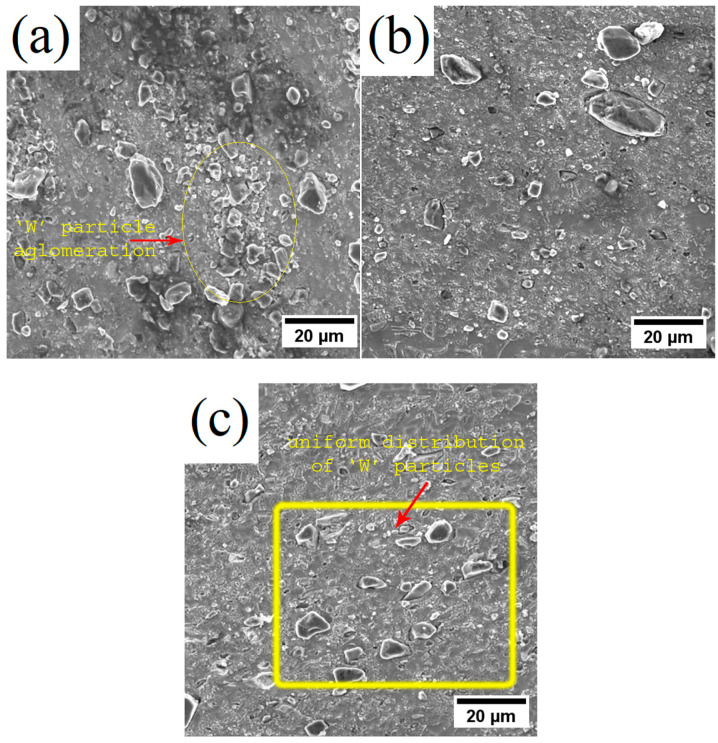
SEM images showing the surface of brass/W prepared at: (**a**) 832 rpm; (**b**) 1000 rpm; (**c**) 1168 rpm.

The surface of brass/W shown in Figure 2a–c indicates a distinct composite microstructure by projecting a uniform dispersion of W in brass. Moreover, W particles do not show any agglomeration or particle-free zones. The selection of suitable process parameters is crucial for producing brass/W composites with a homogeneous distribution of reinforcements. In FSP, generally the most critical parameters that affect the stirring process are the tool rotational and traverse speed. Increasing the rotational speed of the tool produces a homogeneous distribution of reinforcement particles, whereas increasing the traverse speed leads to particle agglomeration. The micrograph images shown in Figure 2a–c indicate that the uniform distribution of W particles is consistent with the findings of Sharma et al. [9], where the increase in tool rotational speed produced a homogeneous distribution.

**Figure 3:**
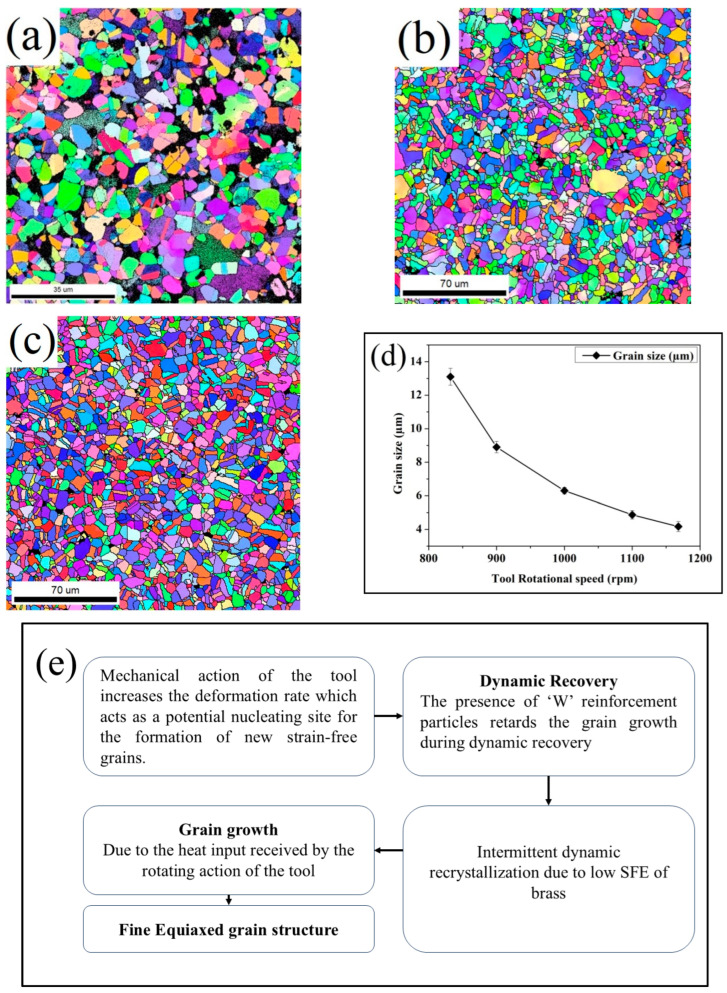
EBSD images depicting brass/W fabricated through FSP at different tool rotational speeds: (**a**) 832 rpm; (**b**) 1000 rpm; (**c**) 1168 rpm; (**d**) grain size distribution; (**e**) mechanism of grain refinement indicating the influence of FSP and addition of W particle.

Figure 3 illustrates the electron backscatter diffraction (EBSD) pattern of as-procured brass and brass/W. The EBSD patterns depict significant changes in microstructure in brass/W surface composites produced under varying tool rotational speeds. The grain size of as-procured brass was determined to be 13.4 µm, indicating a coarser grain structure. In the brass/W, the variations in grain size were observed based on the tool rotational speed. Specifically, when processed at 832 rpm, the composite exhibited a modest reduction in grain size to 13 µm, suggesting that processing conditions influenced grain refinement. The brass/W processed at a higher tool rotational speed of 1168 rpm recorded a lower average grain size value of 4 µm. This reduction in grain size indicates the interdependence of tool rotational speed and grain size. The changes are attributed to factors like deformation, frictional heat, and W particle distribution. The amount of plastic deformation reduces as the rotational speed of the tool reduces and vice versa. The higher tool rotational speed enhances W particle dispersion. The homogeneous distribution causes the formation of a fine-grained microstructure, as reported by Ma [19] and Arora et al. [20]. The incorporation of W particles in brass influences grain growth by stimulating dynamic recovery. Moreover, the uniform dispersion of W particles fosters grain boundary pinning that plays a crucial role in the grain refinement mechanism [20]. Both grain boundary pinning and plastic deformation bestow microstructural change. In addition, the change in grain size is affected by rotational tool speed, which is a critical factor to impact the mechanical properties and wear behavior of brass/W. In line with the above, Sato et al. [21] found a notable change in microstructure and reported a suitable correlation among tool rotational speed, frictional heat, and grain size.

**Figure 4:**
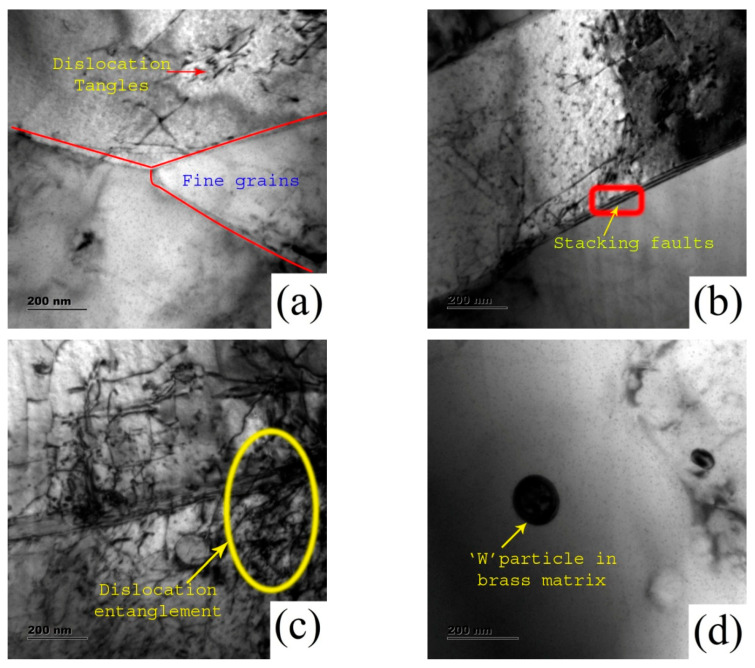
TEM micrographs of brass/W showing: (**a**) fine near-equiaxed grains; (**b**) stacking faults; (**c**) dislocation structures; (**d**) W particle–matrix interface.

The increase in dislocation density in brass/W is attributed to plastic deformation and the frictional heat of FSP. This promotes the rearrangement of dislocations that results in microstructural modification. The tool rotational speed and stirring action during FSP induce plastic deformation, providing room for the generation of strain-free grains. The introduction of W reinforcement in brass restricts the coarsening of grains in the course of dynamic recovery. The thermal input during FSP often surpasses the recrystallization threshold, thereby activating dynamic recrystallization and promoting grain refinement. Dynamic recrystallization occurs in either continuous or discontinuous mode with respect to the stacking fault energy (SFE) of the material. The SFE of brass is low, and it fosters discontinuous dynamic recrystallization [22]. The microstructural modification after FSP follows dynamic recovery, discontinuous dynamic recrystallization, grain coarsening, and the emergence of fine grains [23]. The grain refinement mechanism is shown in Figure 3e. During dynamic recrystallization, nuclei form within plastically deformed regions, and restoration occurs through the migration of grain boundaries. The rearrangement of dislocations contributes to the development of sub-grain boundaries during dynamic recovery, with increasing misorientation that transforms low-angle grain boundaries (LAGBs) into high-angle grain boundaries (HAGBs). The heat input is obtained using the continuous rotational motion of the FSP tool that enhances the grain growth and formation of refined grains. The change in microstructure and W distribution after FSP influences the wear behavior [24]. Figure 4a illustrates the TEM microstructure of the brass/W composite reinforced with 9 vol.% W, characterized by ultrafine grains with dispersed dislocations and localized dislocation-free regions indicating discontinuous dynamic recrystallization. The continuous dynamic recrystallization is restricted in brass/W because of low SFE due to the presence of Zn, and the stacking faults are observed in Figure 4b. As shown in Figure 4c, dislocations are evident within the stir zone due to the combined influence of severe plastic deformation and dynamic recrystallization, while Figure 4d confirms the presence of W particles. Grain refinement during deformation enhances dislocation generation, and thermal mismatch between brass and W induces additional stresses. Furthermore, the large thermal gradients associated with FSP promote lattice slip, leading to a higher dislocation density, as observed in the TEM micrographs of the brass/W composite [21].

### 3.2. Effect of Tool Rotational Speed on Hardness and Wear Behavior

**Figure 5:**
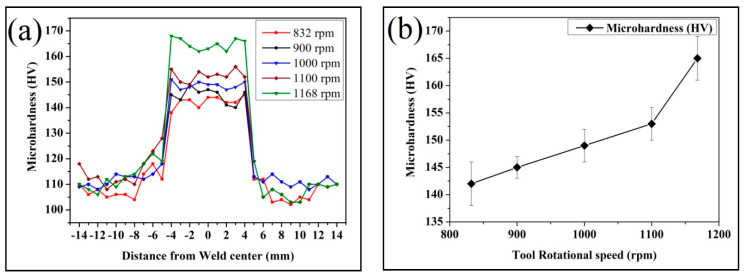
Influence of tool rpm on: (**a**) microhardness distribution; (**b**) average microhardness at the stir zone of brass/W surface composite.

Figure 5a illustrates the effect of tool rotational speed on the microhardness distribution of the brass/W composite across the processed region. A clear increase in hardness is observed near the weld center, particularly within the range of approximately −4 mm to +4 mm, which represents the stir zone (SZ). In this region, the hardness values lie between 145 and 165 HV, whereas the base material away from the processed zone shows lower values, typically around 105 to 115 HV. The regions between these two zones correspond to the thermo-mechanically affected zone (TMAZ), where intermediate hardness values are recorded. Such a distribution is commonly observed in materials subjected to friction stir processing. The higher hardness in the stir zone is due to intense plastic deformation and heat generated during the process, which facilitates dynamic recrystallization and the formation of fine equiaxed grains as recorded in Figure 3a–c. Additionally, the incorporation of W particles strengthens the matrix by restricting dislocation movement, thereby contributing to the overall increase in hardness. It is also noted that the region of maximum hardness aligns with the location of the initially machined keyway, where W particles were introduced. During FSP, these particles are redistributed into the surrounding material due to the stirring action of the tool, resulting in a localized concentration of reinforcement within the stir zone. The improvement can be associated with better material mixing, more uniform W particle distribution and enhanced grain refinement at higher rotational speeds. The nearly symmetrical hardness profile on either side of the weld center further suggests consistent material flow and effective dispersion of the reinforcement during processing. Figure 5b shows the mean hardness within the stir zone. The average hardness increases with increasing tool speed, with the composite fabricated at 832 rpm exhibiting the lowest value (142 Hv) and that produced at 1168 rpm showing the highest hardness (165 Hv). The incorporation of W reinforcement leads to noticeable spatial variations in hardness within the surface composite. The enhanced hardness observed at 1168 rpm is attributed to the improved dispersion of W particles and the formation of finer, equiaxed grains. Higher rotational speeds promote a more uniform reinforcement distribution and increase resistance to dislocation motion. Microstructural factors such as particle dispersion, dislocation density, and grain refinement collectively contribute to the strengthening of the brass/W composite [23]. These observations are consistent with the findings of Sathiskumar et al. [18] who reported variations in microhardness with tool rotational speed in FSP-fabricated copper surface composites.

**Figure 6:**
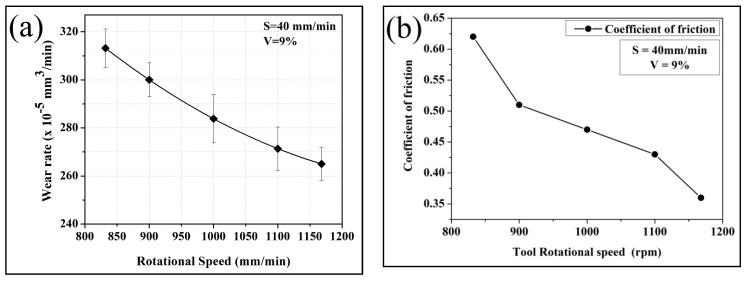
Effect of tool rpm on brass/W: (**a**) wear rate; (**b**) coefficient of friction.

The influence of tool rotational speed on the dry sliding wear behavior of brass/W is presented in Figure 6a, and the corresponding coefficient of friction is shown in Figure 6b. The wear rate decreases with increasing rotational speed, with the highest wear rate recorded for the composite processed at 832 rpm (313 × 10^−5^ mm^3^/m) and the lowest for the sample produced at 1168 rpm (265 × 10^−5^ mm^3^/m). The presence of W reinforcement significantly improves wear resistance by limiting abrasive interactions at the counterface. The observed inverse relationship between wear rate and surface hardness is consistent with Archard’s wear law, wherein increased hardness enhances wear resistance. During sliding, protruding W particles establish load-bearing contact with the counterface, contributing to a reduction in the coefficient of friction, which decreases from 0.62 at 832 rpm to 0.36 at 1168 rpm, as shown in Figure 6b. The coefficient of friction decreases steadily with increasing tool rotational speed. This behavior can be associated with changes in both microstructure and surface interactions during sliding. At higher rotational speeds, the friction stir process enhances the distribution of tungsten (W) particles within the brass matrix. These particles act as hard load-supporting sites at the contact interface, which reduce direct metal-to-metal contact and limit adhesion between the sliding surfaces. In addition, the refined grain structure developed at higher rotational speeds increases the surface hardness, thereby reducing the extent of plastic deformation during sliding. The combined effect of higher hardness and uniform particle dispersion promotes the formation of a stable surface layer at the contact interface, which helps in reducing shear resistance. As a result, smoother sliding conditions are achieved, leading to a lower coefficient of friction. At lower rotational speeds, the relatively poor particle distribution and lower hardness result in greater surface damage, including micro-cutting and ploughing, which increases friction. In contrast, at higher rotational speeds, the wear mechanism shifts toward a more stable adhesive mode with reduced surface damage, contributing to the observed decrease in the coefficient of friction. As indicated by Figure 5b, increasing tool speed enhances hardness while simultaneously reducing wear rate and the coefficient of friction, whereas lower hardness corresponds to reduced wear resistance and greater material loss during sliding [19].

Table 1 presents a quantitative comparison between the present study and previously reported friction stir processed brass and other metal matrix surface composites. It is evident that the present brass/W composite exhibits a significantly refined grain size (~4 µm) and higher hardness (165 HV) compared to similar systems reported in the literature. For instance, brass-based composites reinforced with tungsten and SiC typically show grain sizes in the range of 5–10 µm with hardness values up to ~160–170 HV. These studies on brass and copper-based composites report hardness improvements of 30–40% due to grain refinement and particle strengthening [20,21,22].

**Figure 7:**
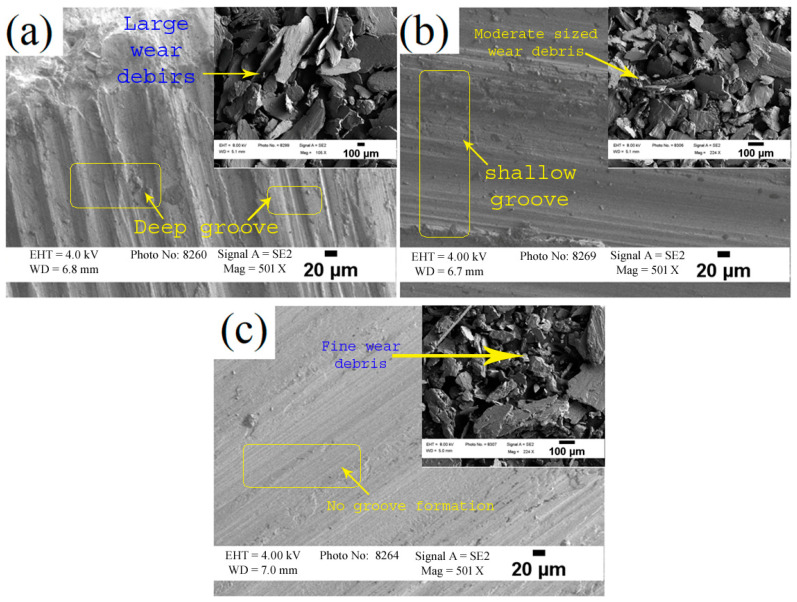
SEM images of brass/W worn surface and wear debris (inset image) produced at: (**a**) 832 rpm; (**b**) 1000 rpm; (**c**) 1168 rpm.

The worn surface of the brass/W developed by FSP with varying tool rpm is represented in Figure 7a–c. In the dry sliding wear test, frictional heat generated by the sliding action softens the surface of the brass/W, resulting in deep cutting marks on brass/W produced at the lowest tool rotational speed (832 rpm). In contrast, the brass/W created at the highest tool rotational speed (1168 rpm) exhibits no deep grooves. For the brass/W produced at 832 rpm (lowest), wear occurred through adhesion as well as micro-cutting, whereas for the one produced at 1168 rpm (highest), the adhesive wear was predominant. The brass/W produced with the lowest rotating tool speed possesses lower hardness, leading to deeper grooves. Conversely, the brass/W created with the fastest tool rotational speed benefits from the uniform distribution of W particles, enhancing its strength and resistance to wear. Furthermore, the improved distribution of W reinforcement reduces the formation of pits on the worn surface. The results of this investigation are in line with the findings of Sathiskumar et al. [18], where the hardness value of the Cu-based surface composite increases with an increase in spindle speed, leading to a lower wear rate.

Figure 7a–c inset depicts the wear debris collected from brass/W after the dry sliding wear test. The wear debris observed in the inset of Figure 7a is large, and it demonstrates a higher wear rate and the severity of cutting at the time of the sliding wear test, resulting in large debris. In contrast, the Figure 7b inset shows wear debris composed of smaller, finer particles. This suggests that the cutting severity did not penetrate deeply into the subsurface, likely due to the uniform dispersion of W reinforcement in brass, which contributed to the formation of smaller debris. As shown in the Figure 7c inset, a further increase in tool rotational speed (produced at 1168 rpm) resulted in a slight decrease in the average size of the wear debris. The presence of W reinforcement in the wear specimen reduces the likelihood of direct contact with the counterface, promoting finer wear debris. The W reinforcements pulled out during sliding contribute to a transition from two-body abrasion to three-body abrasion wear, further facilitating the formation of fine debris. A decrease in the volume fraction of W particles with increased tool rotational speed could potentially reduce the milling action. The above results are in line with the reports of Sathiskumar et al. [18] and Saravanakumar et al. [22], which indicate that higher tool rotational speeds enhance the hardness of surface composites, potentially leading to a lower wear rate in the fabricated surface composite.

## 4. Conclusions

The brass/W produced through FSP by varying tool rotational speeds resulted in the following conclusions:

Microstructural analysis revealed a uniform distribution of tungsten particles within the brass matrix, with no observable clustering. The difference in density between brass and tungsten did not affect particle dispersion. Processing at a traverse speed of 40 mm/min combined with a rotational speed of 1168 rpm resulted in a refined grain structure of approximately 4 µm.

Hardness increased with tool rotational speed, rising from 142 HV at 832 rpm to 165 HV at 1168 rpm. This improvement is attributed to finer grain formation, higher dislocation density, and more effective dispersion of tungsten particles.

Wear behavior was significantly influenced by rotational speed. The sample processed at 1168 rpm exhibited a lower wear rate of 265 × 10^−5^ mm^3^/m, while the sample at 832 rpm showed an approximately 18% higher wear rate. Similarly, the coefficient of friction decreased from 0.62 at 832 rpm to 0.36 at 1168 rpm.

The dominant wear mechanism shifted with processing conditions. At lower rotational speed, abrasive and micro-cutting wear produced coarse debris, whereas at higher rotational speed, adhesive wear dominated, resulting in finer debris and improved surface stability.

Based on the experimental results, the optimal processing condition for the fabrication of brass/W surface composites via friction stir processing was identified as a tool rotational speed of 1168 rpm combined with a traverse speed of 40 mm/min and 9 vol.% tungsten reinforcement. Under these conditions, the composite exhibited a refined grain size, maximum hardness, and lowest wear rate and coefficient of friction. In real-time, specifically, the improved hardness and wear resistance make the developed brass/W surface composites suitable for industrial components subjected to sliding wear, such as bearings, bushes, gears, and automotive or thermal system parts. Furthermore, the ability to enhance surface properties without altering the bulk material offers a cost-effective and efficient surface engineering approach, extending component service life while maintaining core material properties.

## Figures and Tables

**Table 1:** Comparative analysis of the present study and copper-based surface composites fabricated via friction stir processing.

S. No.	Material System	Reinforcement	Key FSP Parameters	Grain Size (µm)	Hardness (HV)	Wear Behavior	Key Findings
1	Brass/W (present study)	W (9 vol.%)	1168 rpm, 40 mm/min	~4 µm	165 HV	265 × 10^−5^ mm^3^/m	Highest hardness & lowest wear due to fine grains and uniform dispersion
2	Brass/W [2]	W (0–18 vol.%)	~1000 rpm (varied)	~5–10 µm	~150–170 HV	Improved wear resistance	Uniform dispersion without agglomeration; hardness increases with W fraction
3	Brass/SiC [25]	SiC (0–18 vol.%)	1600 rpm, 60 mm/min	Significant refinement (≤5 µm)	~160 HV (approx.)	Improved wear resistance	Dynamic recrystallization and uniform SiC dispersion enhanced tribology
4	Brass/carbon fiber [26]	CF (varied)	Variable speed	Refined grains	Increased hardness	Improved wear	Reinforcement fraction strongly influences microstructure and properties
5	Cu-based composites [27]	Various particles	Depends on rpm & speed	Fine grains (~1–10 µm)	Improved hardness	Reduced wear	FSP enables uniform particle dispersion and grain refinement

## Data Availability

The original contributions presented in this study are included in the article. Further inquiries can be directed to the corresponding author.
